# Supplementary material for: Epidemiology of soil transmitted helminths and risk analysis of hookworm infections in the community: Results from the DeWorm3 Trial in southern India
Source: PLoS Negl Trop Dis. 2021 Apr 30;15(4):e0009338. doi: 10.1371/journal.pntd.0009338 (PMC8184002; doi:10.1371/journal.pntd.0009338)
Supplement: S1 Table — (DOCX) [file pntd.0009338.s002.docx]

**S1 Table: Comparison of characteristics of those consenting and refusing participation in the longitudinal monitoring cohort**

|  | **Selected individuals from the census (10,144)**  **n (%)** | **Individuals consenting to participate in LMC (6503)**  **n (%)** | **Individual refusals to participate in LMC (274)**  **n (%)** | **LMC participants who provided sample (6092)**  **n (%)** | **LMC participants who refused to provide a sample (411)**  **n (%)** |
| --- | --- | --- | --- | --- | --- |
| **Age category** |  |  |  |  |  |
| PSAC (1 - 4 years) | 1942 (19) | 1242 (19) | 23 (8) | 1179 (19) | 63 (15) |
| SAC (5 - 14 years) | 2096 (21) | 1364 (21) | 34 (12) | 1305 (21) | 59 (14) |
| Adult (15+ years) | 6106 (60) | 3897 (60) | 217 (79) | 3608 (59) | 289 (70) |
| **Sex** |  |  |  |  |  |
| Male | 5071 (50) | 3045 (47) | 122 (45) | 2838 (47) | 207 (50) |
| Female | 5072 (50) | 3457 (53) | 152 (55) | 2353 (53) | 204 (50) |
| **SES (1=High, 5=Low)** |  |  |  |  |  |
| 1 | 2363 (23) | 1472 (23) | 104 (38) | 1378 (23) | 94 (23) |
| 2 | 2310 (23) | 1516 (23) | 61 (22) | 1425 (23) | 91 (22) |
| 3 | 2017 (20) | 1329 (20) | 44 (16) | 1251 (21) | 78 (19) |
| 4 | 1836 (18) | 1185 (18) | 36 (13) | 1101 (18) | 84 (20) |
| 5 | 1617 (16) | 1001 (15) | 29 (11) | 937 (15) | 64 (16) |
| **Education - head of the household** | |  |  |  |  |
| No education | 3140 (31) | 1962 (30) | 70 (26) | 1836 (30) | 126 (31) |
| Some primary | 1863 (18) | 1202 (18) | 47 (17) | 1121 (18) | 81 (20) |
| Some middle | 1929 (19) | 1270 (20) | 49 (18) | 1187 (19) | 83 (20) |
| Some secondary | 2062 (20) | 1341 (21) | 56 (20) | 1268 (21) | 73 (18) |
| Some higher secondary / college | 1067 (11) | 675 (10) | 50 (18) | 634 (10) | 41 (10) |
| **Family size** |  |  |  |  |  |
| <=4 members | 4827 (48) | 3098 (48) | 140 (51) | 2901 (48) | 197 (48) |
| >=5 members | 5317 (52) | 3405 (52) | 134 (49) | 3191 (52) | 214 (52) |
| **Sanitation** |  |  |  |  |  |
| Improved | 3583 (35) | 2250 (35) | 115 (42) | 2122 (35) | 128 (31) |
| Unimproved facilities | 6560 (65) | 4253 (65) | 159 (58) | 3970 (65) | 283 (69) |
