# Supplementary material for: Epidemiology of soil transmitted helminths and risk analysis of hookworm infections in the community: Results from the DeWorm3 Trial in southern India
Source: PLoS Negl Trop Dis. 2021 Apr 30;15(4):e0009338. doi: 10.1371/journal.pntd.0009338 (PMC8184002; doi:10.1371/journal.pntd.0009338)
Supplement: S2 Table — (DOCX) [file pntd.0009338.s003.docx]

**S2 Table: Prevalence of other helminths that were detected by Kato Katz (n=6089)**

| **Other helminth species** | **n (%)** |
| --- | --- |
| *Strongyloides* spp. (larvae) | 1 (<0.1) |
| *Enterobius vermicularis* | 96 (1.6) |
| *Taenia* spp. | 1 (<0.1) |
| *Hymenolepis nana* | 27 (0.4) |
| *Hymenolopis diminuta* | 6 (0.1) |
| *Cappillaria* ova | 1 (<0.1) |
| Total | 132 (2.2) |
