# Supplementary material for: Epidemiology of soil transmitted helminths and risk analysis of hookworm infections in the community: Results from the DeWorm3 Trial in southern India
Source: PLoS Negl Trop Dis. 2021 Apr 30;15(4):e0009338. doi: 10.1371/journal.pntd.0009338 (PMC8184002; doi:10.1371/journal.pntd.0009338)
Supplement: Attachment — Submitted filename: PNTD-D-20-01998 LC.docx [file pntd.0009338.s004.docx]

General comments:

This is a well-written description and analysis of baseline results from the Indian study sites of the Deworm3 study. I only have some questions for clarification and a small suggestion for the predictors considered in the analyses.

Methods:

Please clarify how the reader should interpret the following: “All samples were screened in duplicate by the Kato-Katz method by trained technicians who screened each slide (also labelled with the QR code sticker) for a minimum of six to eight minutes and within 30 minutes of preparation. If any of the three STH species were present, the number of eggs in each slide was recorded on phones also using SurveyCTO software-based forms.” I have never seen KK being described like this. Should this be interpreted as an initial screen followed by the actual counting? The use of an initial screening procedure could theoretically generate excess zero counts compared to an approach where all microscope fields are examined. Also, in an earlier paper describing data from this region (and I presume the same study?), it was described that a saline and iodine wet preparation was done as an initial screen, and that only positive samples were processed with KK (<https://doi.org/10.1016/j.conctc.2016.12.002>). If that was done in the current study as well, it should be clearly described, as such an approach has been shown to introduce excess zero counts which should be kept in mind when comparing results to “pure” KK (<https://doi.org/10.1093/infdis/jiz535>).

The authors perform mixed-effects negative binomial regression to model the egg intensities. Because mixed-effects NB regression is notoriously fickle when it comes to model convergence, can the authors confirm that all the presented models converged, and if not, what steps were taken to try and achieve convergence?

If possible, I suggest that the authors explore one additional predictor: the proportion of the cluster population reporting having taken deworming. The deworming variable was accounted for at the individual-level, but because deworming – at high enough coverage – also has an effect on transmission in a community, it should also be considered at that level.

Discussion:

The authors write “A recent study including a WASH intervention, however, did not show any additional benefit over and above a deworming intervention across all age groups (Nery 2019) highlighting the importance of more comprehensive behavioural and structural WASH interventions and access required.” Although I agree with the point that behavioural and structural WASH interventions and access are required, using the Wash4Worms study as an argument for this is not entirely valid as that study was not even able to show an effect because of the way it was designed (any potential WASH impact was masked by the effect of community-wide deworming), as explained and illustrated in a linked modelling study (<http://dx.plos.org/10.1371/journal.pntd.0006758>).

I’m not sure I understand why a geospatial analysis is not be possible because of the trial’s blinding restrictions.

Minor comments:

- Introduction
  - Not sure if this is intentional, but the year in brackets doesn’t match the year in the main sentence: “…Global Burden of Disease estimates, 2013 (GBD 2015).”
- Methods
  - What is a “schedule tribe” (in reference to the Malayali tribe)?
- Table 3:
  - Rephrase the subheader (first column): “Highest education level of the any female member of the family”
