# Supplementary material for: Epidemiology of soil transmitted helminths and risk analysis of hookworm infections in the community: Results from the DeWorm3 Trial in southern India
Source: PLoS Negl Trop Dis. 2021 Apr 30;15(4):e0009338. doi: 10.1371/journal.pntd.0009338 (PMC8184002; doi:10.1371/journal.pntd.0009338)
Supplement: Attachment — Submitted filename: India_R1_PNTD Responses to reviewers_04Mar2021.docx [file pntd.0009338.s005.docx]

**RESPONSE TO REVIEWERS**

***PNTD-D-20-01998: Epidemiology of soil transmitted helminths and risk analysis of hookworm infections in the community: results from the DeWorm3 Trial in southern India***

**METHODS**

**Reviewer #1**

The objectives and study design are clear. The setup seems appropriate and thorough, and the aims are reasonable given the study design. The statistical methods appear appropriate and ethical requirements appear to have been followed.

Please provide more info about how you went from 10,144 to 8517. It’s not clear to me from Figure 2 either—what made the additional people “not required”?

*The total sample size for the longitudinal monitoring cohort was determined a priori (6000 individuals - 150 individuals from each of the 40 clusters with an age distribution of 30 PSAC, 30 SAC and 90 adults). Following the baseline census at the 3 sites, 150 sampled individuals stratified by age group were identified. All these individuals were required to be visited, up to three times (if not present on the first visit). However, given that not all participants were available or willing to participate, a stage-two list of 75 reserve/replacement participants, age-stratified and also ranked, was released once a status was recorded for each of the initial 150 sampled individuals per cluster. An individual unavailable/unwilling to participate, could be replaced with the first ranked individual from the reserve list from the same age group. A stage-three reserve list was released if the original and stage-two lists were exhausted without full recruitment in the three age groups in a given cluster. This resulted in an excess number of individuals from the reserve lists who were eligible but were not approached as the full sample for the cohort was achieved. We have now added a footnote to the figure.*

*Page 14, Figure 2 Footnote, Lines 563 — 566:*

*“Footnote: “Not required” refers to the number of individuals in the ranked, age-stratified, cluster-specific reserve/replacement lists generated who were not approached as the cohort sample size was achieved in the cluster”*

Were surveys undertaken entirely in Tamil, or were any tribal or other languages used?

*The surveys were entirely in Tamil. The tribal communities in Jawadhu Hills also speak Tamil, not any other language. This is now mentioned in the methods section.*

*Page 6, lines 211 – 215:*

*“The survey was conducted between December 2017 to February 2018 in local language spoken by all participants (Tamil) during which individual-level data including school enrolment, highest education level achieved, deworming history and shoe-wearing at the time of the survey were collected.”*

**Reviewer #2**

Yes. This study is nested within a much larger, very well-resourced and very well designed, longitudinal cohort study. It would improve with further clarity on the stool specimens collected:

Since the predominant parasite identified is hookworm, which has the least in vitro survival (once the stool is voided, from amongst the other STH parasites), and the key study outcome is intensity or number of hookworm eggs per gram of stools, it is very important to share the time from stool voiding to reading the slides. A delay can falsely underestimate the intensity. It is mentioned that stools were collected daily and transferred for lab reading every day: not clear how that translates to individual sample read time (median/mean and range, etc.).

*We agree with the reviewer that a delay in reading the slide from the time stool was collected could result in loss or decay of hookworm eggs. The median (SD) time taken for the Kato Katz slide to be read from the time of sample collection was 5.4 (2.3) hours with minimum of 1 hour and maximum of 12.7 hours for 5171 samples. This period includes the uniform waiting time of 30 minutes before screening after preparation of the smear.*

**Reviewer #3**

Please clarify how the reader should interpret the following: “All samples were screened in duplicate by the Kato-Katz method by trained technicians who screened each slide (also labelled with the QR code sticker) for a minimum of six to eight minutes and within 30 minutes of preparation. If any of the three STH species were present, the number of eggs in each slide was recorded on phones also using SurveyCTO software-based forms.” I have never seen KK being described like this. Should this be interpreted as an initial screen followed by the actual counting? The use of an initial screening procedure could theoretically generate excess zero counts compared to an approach where all microscope fields are examined. Also, in an earlier paper describing data from this region (and I presume the same study?), it was described that a saline and iodine wet preparation was done as an initial screen, and that only positive samples were processed with KK (https://doi.org/10.1016/ j.conctc.2016.12.002). If that was done in the current study as well, it should be clearly described, as such an approach has been shown to introduce excess zero counts which should be kept in mind when comparing results to “pure” KK (https://doi.org/10.1093/infdis/jiz535).

*We clarify that the Kato Katz slide was examined and the number of eggs were recorded if there were any. No saline iodine preparations were made in this study. The paper referred to by the reviewer is a previous study not Deworm3 related. We have now modified the statement slightly.*

*Page 6, Lines 223 – 227:*

*“All samples were read in duplicate by the Kato-Katz method by trained technicians who screened each slide (also labelled with the QR code sticker) for a minimum of six to eight minutes and within 30 minutes of preparation. The number of eggs in each slide was recorded on smartphones also using SurveyCTO software-based forms.”*

The authors perform mixed-effects negative binomial regression to model the egg intensities. Because mixed-effects NB regression is notoriously fickle when it comes to model convergence, can the authors confirm that all the presented models converged, and if not, what steps were taken to try and achieve convergence?

*We clarify that the models presented converged and the methods to achieve this have been explained – we refer to Page 7, Lines 302 to 306:*

*“Since STHs are highly aggregated and distributed in a negative binomial manner, the association between the intensity (EPG) of infection and associated factors was analysed using mixed-effects negative binomial regression method of the egg counts offsetting the actual quantity of stool used per sample and accounting for the clustering at all the levels.”*

If possible, I suggest that the authors explore one additional predictor: the proportion of the cluster population reporting having taken deworming. The deworming variable was accounted for at the individual-level, but because deworming – at high enough coverage – also has an effect on transmission in a community, it should also be considered at that level.

*We thank the reviewer for this insightful comment. In our analysis, although deworming is accounted at the individual level, the household, village and cluster levels were included in the multilevel multivariable logistic regression model and the model estimate obtained for the deworming variable was adjusted at the cluster level (methods section page 7, Lines 300 — 302).*

*Regarding the comment on cluster level deworming, prior to the conduct of DeWorm3 MDA, deworming was delivered primarily through the NDD, targeting children aged 1-19 years and for these, we do not have cluster- or village-level coverage data for the NDD programme as government data was collected only at the school level. We do not expect significant variability in the school based coverage reported at this level. Estimating a cluster-level deworming coverage variable on the basis of this stratified sample of 150 individuals per cluster may not provide a robust variable for inclusion. However, we agree this is a very valid point and we will explore the effect of community-level deworming coverage and transmission in a future analysis with the data collected from this trial.*

**RESULTS**

**Reviewer #1**

The results are clear, reasonable, and match the analysis plan. The figure and tables are appropriate and interesting. The image resolution appears a bit low, but perhaps will be fine if they are not shown in this large size in the final version.

Your main findings in terms of correlates of hookworm infection seem to be environmental (though you acknowledge that this may be affected by environmental differences between the study sites), SES/education, and history of deworming. You use a mixture of univariable and multivariable models, but I would like to know whether history of deworming and SES/education are correlated. Two potential ways I could see this occurring are if a) people with more education had better access to healthcare or b) people with more education were more aware of past deworming as they had been provided with information about past treatment in a way that was accessible to them. If they are correlated with each other, it might be worth noting, just as you mention how you controlled for other variables when looking at social determinants. If not, then no issues.

*The Pearson correlation coefficients for i) history of deworming vs education of the head of the household was 0.065, ii) history of deworming vs socio-economic status was 0.05 and iii) education of the head of the household vs socio-economic status was 0.35 - indicating weak correlations. To further explore this, we also created a Pearson’s correlation coefficient matrix for history of deworming, socio-economic status and education of the head of the household with all the categories in those variables and found similar results (Table below). Finally, the three variables remained significant in the multilevel multivariable logistic regression model indicating that they are independently associated with hookworm prevalence.*

*Note: The correlation matrix was estimated post multilevel model so the reference categories were omitted. Reference categories for each were: Deworming - No, Education - No education, and SES - Quintile 1*

**Reviewer #2**

Excellent and clearly presented results linked to the study objective. Minor question below can be clarified:

1. Authors report a higher refusal rate in persons from higher SES and education status (page 13 - Results). Could this translate to possible overestimation of the hookworm prevalence?

*Although low SES and education (of the head of household) were reported to be associated with increased hookworm prevalence, we do not anticipate a significant overestimation of hookworm prevalence as the proportion of individuals refused was only 4.5%. To further explore whether these refusals affected the prevalence of 17% (95% CI: 16 - 17.9), we performed adjusted estimates with 3 scenarios assuming all 274 individuals who refused to participate in each of the SES, education and sanitation categories: i) were all positive for hookworm (20%) ii) were all negative for hookworm (16%) and iii) had the same prevalence rates of persons who provided stool sample (17%) and found no significant change.*

2. What was the reported PCT coverage for previous LF MDAs in the study areas? Would be good to know to relate to possible impact on Ascaris and Trichuris (though latter is less impacted by Albendazole treatment alone).

*The study sites selected as a prerequisite for Deworm3 were LF STOP districts. Vellore district received its last round of LF MDA in 2013 and Thiruvanamalai in 2015. We have included this in the methods section on study setting.*

*Page 5, Lines 155-156:*

*“The last round of LF MDA was carried out in Vellore district in 2013 and Thiruvanamalai in 2015. (Elango, 2018).”*

*The LF MDA coverage survey conducted in the state of Tamil Nadu in three districts for the two rounds of MDA (2009 and 2010) reported an average coverage and consumption of tablets as 76.3% and 51.7% respectively among the sampled population. Data at the district level for Vellore and Thiruvanamalai were not available.*

*Reference: Nandha B, Krishnamoorthy K, Jambulingam P. Towards elimination of lymphatic filariasis: social mobilization issues and challenges in mass drug administration with anti-filarial drugs in Tamil Nadu, South India. Health Educ Res. 2013 Aug;28(4):591-8.*

**DISCUSSION**

**Reviewer #3**

The authors write “A recent study including a WASH intervention, however, did not show any additional benefit over and above a deworming intervention across all age groups (Nery 2019) highlighting the importance of more comprehensive behavioral and structural WASH interventions and access required.” Although I agree with the point that behavioral and structural WASH interventions and access are required, using the Wash4Worms study as an argument for this is not entirely valid as that study was not even able to show an effect because of the way it was designed (any potential WASH impact was masked by the effect of community-wide deworming), as explained and illustrated in a linked modelling study (<http://dx.plos.org/10.1371/journal.pntd.0006758>).

*We have modified the statement to illustrate that while measuring impact of WASH is complicated, the importance of integrating WASH in the longer term is necessary.*

*Page 12, Lines 500- 504:*

*“While the effect of WASH interventions are not easily evaluated especially in the context of other interventions (Nery 2019), the importance of integrating comprehensive behavioral and structural WASH interventions and access to potentially sustain the gains made from deworming in the longer term has been highlighted in a recent modelling study (Coffeng, 2018).”*

I’m not sure I understand why a geospatial analysis is not be possible because of the trial’s blinding restrictions.

*The Deworm3 Trial Data Safety Monitoring Board (DSMB) have instructed that to ensure blinding, disaggregated data (at the cluster level) including any geospatial analysis cannot be reported before the end of the study.*

**Conclusions**

**Reviewer #1**

The conclusions are well-supported, limitations are described (perhaps a bit more on potential bias especially in terms of the cascade of how participants were selected or opted out all the way down from the census to the number in the end with parasitological data). Good implications and relevance. Perhaps address any concerns if there's any chance (mentioned above in results comments) that people with lower educational attainment are less likely to report recent deworming (or that these two variables are potentially related in any other way).

*As detailed in the response to comments on the results section, socioeconomic status, education (of head of household) and history of deworming were not correlated and remained as significant in the multilevel multivariable logistic regression model.*

**MINOR MODIFICATIONS**

**Reviewer #1:**

Since biannual can mean either twice a year or once every two years, please clarify by changing to “twice a year”

*We have reworded the abstract, author summary, and introduction accordingly.*

*Abstract, Page 2, Lines 31 — 32:*

*“Since 2015, India has coordinated the largest school-based deworming program globally, targeting soil-transmitted helminths (STH) in ~250 million children aged 1 to 19 years twice yearly.”*

*Author Summary, Page 3, Lines 64 — 67:*

*“To tackle this large burden of infection, the government of India launched one of the world’s largest school-based deworming programs in 2015 aiming to deworm all pre-school and school-aged children between 1 to 19 years of age twice yearly on the National Deworming Days.”*

*Introduction, Page 4, Lines 105 —109:*

*“The Ministry of Health and Family Welfare (MOHFW) in India has since introduced the world’s largest school-based deworming program, targeting ~240 million children aged 1 to 19 years twice yearly (biannual) during the ‘National Deworming Days’ (NDD) conducted in February and August since 2015 (National Deworming Day, 2020).”*

I think Lo 2018 might be better summarized as something like “a study using data from 45 countries found that there was a consistent association between deworming and reduced stunting in pre-school-age children (PSAC)” rather than “multiple studies have shown the benefits of deworming in endemic countries including a reduction in stunting in pre-school-age children (PSAC)”

*The sentence has been modified as suggested.*

*Page 4, Lines 91 — 95:*

*“While a recent Cochrane review indicated that regular deworming of children in public health programmes does not seem to improve outcomes (Taylor-Robinson 2019),* *a study using data from Demographic and Health Surveys (DHS) of 45 STH endemic countries found that there was a consistent association between deworming and reduced stunting in pre-school-age children (PSAC) (Lo 2018)”*

Perhaps you could define an anganwadi centre.

I quibble with calling school-based deworming a “highly effective approach”, as it might be interpreted as meaning highly effective at reducing infection, which is the subject of your study rather than the basis for it. You might rephrase to “this is a highly effective way of reaching PSAC and SAC”.

*The definition of an “angandwadi centre” has been included and the sentence on school-based deworming strategy has been modified as suggested.*

*Page 4, Lines 110 — 115:*

“*With primary school enrolment exceeding 99% in India (ESAG, 2018) and the involvement of anganwadi centers (a government run centre in each village providing care for pregnant women and children under 6 years of age under the Integrated Child Development Services Scheme), this is a highly effective way of reaching out to PSAC and SAC to carry out a targeted deworming program.”*

**Reviewer #3**

Introduction: Not sure if this is intentional, but the year in brackets doesn’t match the year in the main sentence: “…Global Burden of Disease estimates, 2013 (GBD 2015).”

*The estimates are for the years 1990-2013 while the manuscript was published as a group authorship in 2015, hence the mismatch.*

Methods: What is a “schedule tribe” (in reference to the Malayali tribe)?

*The sentence has been modified to include the definition of a scheduled tribe.*

*Page 5, Lines 166 — 168:*

*“The tribal or indigenous people that populate this area are the Malayali and are classified as a scheduled tribe (disadvantaged communities or group of people listed in a schedule of the Indian constitution under article 342) by the government.”*

Table 3: Rephrase the sub-header (first column): “Highest education level of the any female member of the family”

*This has been rephrased.*
